# The Impact of the Epigenetic Cancer Drug Azacitidine on Host Immunity: The Role of Myelosuppression, Iron Overload and *tp53* Mutations in a Zebrafish Model

**DOI:** 10.3390/cancers11091294

**Published:** 2019-09-02

**Authors:** Shu-Ching Wang, Ching-Tse Wu, Dong-Yu Wu, Caleb Gon-Shen Chen, Kuo-Ming Chang, Chien-Chung Chang

**Affiliations:** 1 Institute of Molecular and Cellular Biology, National Tsing Hua University, Hsinchu 30013, Taiwan; 2 Department of Hematology, Mackay Memorial Hospital, Taipei 10449, Taiwan; 3 Department of Pathology and Laboratory Medicine, Hsinchu Mackay Memorial Hospital, Hsinchu 35071, Taiwan; 4 Department of Life Science, National Tsing Hua University, Hsinchu 30013, Taiwan

**Keywords:** azacitidine, myelodysplastic syndromes, myelosuppression

## Abstract

The unsatisfactory real-world efficacy of the hypomethylating agent azacitidine in treating myelodysplastic syndromes (MDS) and acute myeloid leukemia (AML) has prompted us to investigate the hematological adverse events and host variables that may compromise the use of this epigenetic drug. Using the zebrafish, we found that azacitidine destroyed their myeloid precursors and impaired myeloid function by inhibiting antigen processing, allogeneic response and phagocytic activity, resulting in increased susceptibility to infection even by the normal flora *E. coli*. In addition, iron overload, a MDS-associated condition following repeated transfusions, exacerbated bacterial infection especially by *V. vulnificus* with known iron dependence. Furthermore, we show that the *tp53^M214K^* mutant zebrafish survived longer than the wild-type (WT) when challenged with bacteria following azacitidine treatment. This was attributed to the mutant’s hematopoietic cells rather than its general genetic background, since the WT animals reconstituted with the *tp53^M214K^* mutant kidney marrow became more resistant to bacterial infection following treatment with azacitidine. The clinical relevance of our findings was indicated by a MDS case with severe azacitidine-induced bone marrow suppression and by the association of hyperferritinemia with bacteremia in azacitidine-treated patients, while *tp53^M214K^*-mediated resistance to azacitidine-induced myelosuppression may explain the survival advantage of malignant MDS and AML clones over their normal counterparts under azacitidine treatment. Together, we propose that myelosuppression, iron overload and *TP53* mutations may represent the host variables that compromise the azacitidine efficacy.

## 1. Introduction

Epigenetic alterations occur frequently in human cancers and play an important role in driving the cancer phenotype [1]. These alterations include DNA methylation, histone modifications and their interplay that aberrantly silence or induce genes involved in how cancers develop, grow, evade the host immune system and metastasize [2]. One classic example is silencing of tumor suppressor genes (TSGs), such as *MLH1* [3], which encodes a DNA mismatch repair enzyme that functions as a brake to halt cancer evolution. Another example is silencing of genes responsible for myeloid stem cell differentiation, resulting in ineffective hematopoiesis and malignant clonal expansion, such as in myelodysplastic syndromes (MDS) and its subsequent transformation to acute myeloid leukemia (AML) [4]. Gene silencing is often mediated by DNA methyltransferases (DNMTs), which add a methyl group to the cytosine (5-mC) in the CpG islands of a gene promoter, leading to hypermethylation and promoter inactivation [1]. This hypermethylated state can also be strengthened by mutations or deregulation of the 5-mC modifier, i.e., ten–eleven translocation methylcytosine dioxygenase (TET) enzymes [1]. 

The dependence of disrupted epigenetic machinery varies among different cancer types. In MDS, aberrant DNA hypermethylation is a dominant mechanism for disease progression [5]. This finding provided a rationale to develop demethylating agents capable of reactivating silenced genes to promote differentiation and/or restore the function of TSGs in malignant hematopoietic stem cells in MDS and AML. 

Among all the demethylating or hypomethylating agents (HMAs), azacitidine (5-azacytidine), a cytidine analog, and its deoxyribose counterpart decitabine (5-aza-2′-deoxycytidine), were approved by FDA in 2004 and 2006, respectively, to treat MDS and AML [6]. The proposed mechanism of action of azacitidine somewhat differs from that of decitabine in that the former inhibits DNMTs in addition to interrupting RNA synthesis, whereas the latter can only block DNMTs. Alternatively, both drugs can induce a DNA damage response when incorporated into DNA, resulting in apoptosis [1]. However, despite the impressive median overall survival (OS) prolongation of the azacitidie group vs. conventional care group (24.5 months, vs. 15.0 months, *p* = 0.0001) as reported in the randomized phase III AZA-001 trial [7], the real-world experiences with azacitidine for treating MDS and AML have been unsatisfactory [8,9,10]. In a study analyzing 1101 patients (825 MDS; 276 AML) receiving azacitidine, the median OS was found to be 11.6 months [9]. This discrepancy raised the question of whether uncharacterized host factors or adverse events could have limited the efficacy of this epigenetic drug, thus highlighting the pressing need to address these questions in more detail in preclinical models.

In this study, we tested whether the zebrafish, a model organism with a human-like hematopoiesis system [11], could serve as a tool to investigate the hematological adverse effects of azacitidine on host immunity in conjunction with other host variables. We found that this drug could induce profound myelosuppression, leading to impaired antigen processing, allogeneic response, and phagocytic activity, resulting in high vulnerability to bacterial infection, which was exacerbated by iron overload. Intriguingly, we also revealed that the *tp53^M214K^* mutation carried by the zebrafish hematopoietic cells could mitigate azacitidine-induced myelosuppression by preserving their numbers and phagocytic function, thus reducing the host’s susceptibility to bacterial infection. This finding may explain why the *TP53* mutant malignant MDS myeloid stem cells can have a survival advantage over the normal counterparts in patients under azacitidine treatment.

## 2. Results

### 2.1. Marked Myelosuppression Iuduced by Azacitidine in Zebrafish

**Figure 1:**
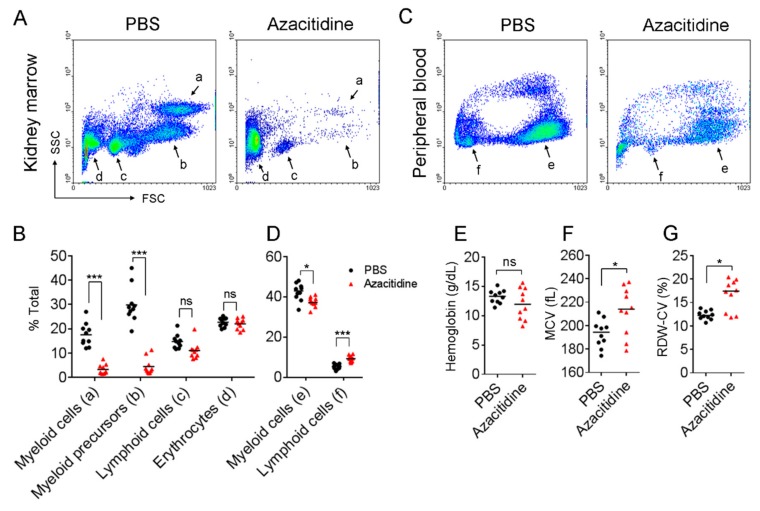
Marked myelosuppression induced by azacitidine in zebrafish. Dot-plot representation of (**A**) whole kidney marrow (WKM) and (**B**) peripheral blood cells isolated from phosphate-buffered saline (PBS)- and azacitidine-treated zebrafish analyzed by flow cytometry. The relative percentage of subpopulations as indicated were measured and presented as median in (**C**,**D**); each dot in the plot represents the percent gated area; *n* = 10. The hemoglobin concentration (**E**), mean corpuscular volume (MCV) (**F**) and red cell distribution width-coefficient of variation (RDW-CV) (**G**) were measured by an automated blood analyzer. *n* = 10. * *p* < 0.05, *** *p* < 0.001, ns, not significant, by the Mann–Whitney *U* test.

One prominent side effect associated with azacitidine therapy in patients is neutropenia [12]. To investigate whether azacitidine suppressed myeloid hematopoiesis in zebrafish, we treated the animals with this drug (3 μg at 10 mg/mL per animal) via intraperitoneal (i.p.) injection once a day for three consecutive days, and then analyzed their kidney marrow (equivalent to bone marrow in mammals) and peripheral blood hematopoietic cell subpopulations on day 4 by flow cytometry. Zebrafish injected with phosphate-buffered saline (PBS) were analyzed in parallel as the mock-treated counterparts. Figure 1A shows that following three daily administrations, multiple hematopoietic cell subsets were suppressed in azacitidine-treated animals, compared with their mock-treated controls. The median percentage of myeloid cells and myeloid precursors were significantly reduced by 86.7% (16.69% vs. 2.22%, *p* < 0.001) and 88.95% (28.5% vs. 3.15%, *p* < 0.001), respectively (Figure 1B), while the percentage of lymphoid cells and erythrocytes were not significantly affected between the test and control groups (Figure 1B).

In the peripheral blood, the myeloid cell subset in azacitidine-treated zebrafish was also reduced by 13.2% (42.95% vs. 37.3%, *p* < 0.05), whereas the lymphoid cells increased by 72.9% (5.35% vs 9.25%, *p* < 0.001) (Figure 1C,D). Intriguingly, the size of lymphoid cells in the azacitidine group increased by ~1.5-fold (forward scatter (FSC) 321.3 ± 43.2 vs. 221.7 ± 17.5, *p* < 0.05), suggesting their activation status. On the other hand, erythrocytes in azacitidine-treated animals showed increased median mean corpuscular volume (MCV) (194.4 fL vs. 214.0 fL, *p* < 0.05) (Figure 1F) and median red cell distribution width-coefficient of variation (RDW-CV) (12.25% vs. 17.45%, *p* < 0.05) (Figure 1G), while had a marginally lower hemoglobin level (13.3 g/dL vs. 11.95 g/dL, *p* = 0.39) that did not reach statistical significance (Figure 1E). These results indicate that azacitidine causes neutropenia with a macrocytic anemia-like symptom in zebrafish.

**Figure 2:**
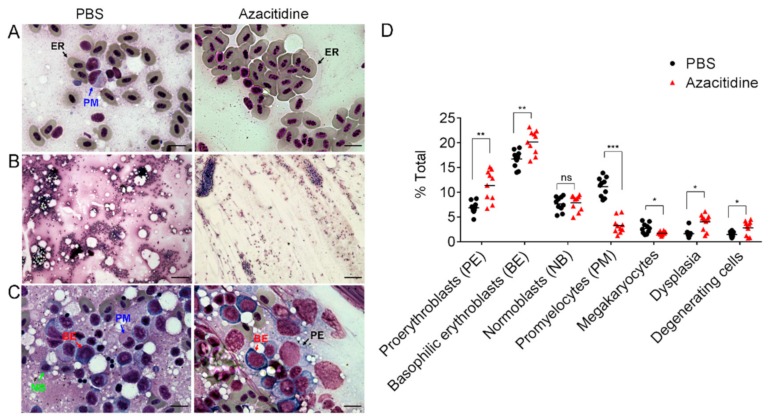
Marked suppression of myeloid series in WKM of zebrafish treated with azacitidine. Liu’s stained micrographs of PBS- and azacitidine-treated peripheral blood (**A**) and kidney marrow smears (**B**,**C**). Quantifications of indicated subpopulations are shown in (**D**); each dot in the plot represents the average of relative cell counts under 10 high-power fields for one WKM smear; *n* = 10 for each group. ER, erythrocyte; * *p* < 0.05, ** *p* < 0.01, *** *p* < 0.001, ns, not significant, by the Mann–Whitney *U* test. Scale bars in (**A**) and (**C**), 10 μm; scale bar in (**B**), 100 μm.

Using histochemical staining, we further examined the morphology of peripheral blood and kidney marrow hematopoietic cell subsets under a light microscope. Figure 2A shows a reduced nuclear/cytoplasm (N/C) ratio of erythrocytes in the peripheral blood of azacitidine-treated zebrafish, compatible with their increased MCV. In the kidney marrow smears, under low-power (4× objective) magnification (Figure 2B), there is pronounced hypocellularity with fibrotic changes in the azacitidine group. Under high-power (100× objective) magnification (Figure 2C), two of the three identified erythroid precursor subsets, namely proerythroblasts (PE) and basophilic erythroblasts (BE), were found to have a median increased percentage (6.88% vs. 11.33%, *p* < 0.01; 16.7% vs. 20.18%, *p* < 0.01) by 1.65-fold and 1.2-fold, respectively, in azacitidine-treated animals compared with mock-treated controls (Figure 2D). On the other hand, the number of promyelocytes (PM) (myelocytic precursors) was markedly reduced in the azacitidine group (median 11.14% vs. 3.25%, *p* < 0.001) (Figure 2D). In addition, there was also a decrease in megakaryocyte numbers in the azacitidine group (median 4.11% vs. 2.30%, *p* < 0.05), suggesting a negative effect on megakaryopoiesis. Notably, there were also more dysplastic and degenerating cells (1.64% vs. 4.02%, *p* < 0.05 and 1.47% vs. 2.83%, *p* < 0.05, respectively) observed in the azacitidine group (Figure 2D). Together, these results suggest that following azacitidine treatment, the development of zebrafish myeloid cells is markedly suppressed probably through a cytotoxic effect, with a relatively increased percentage or a likely compensatory development of early-stage erythroid precursors.

### 2.2. Impaired Antigen Processing and Allogeneic Rejection in Azacitidine-Treated Zebrafish

**Figure 3:**
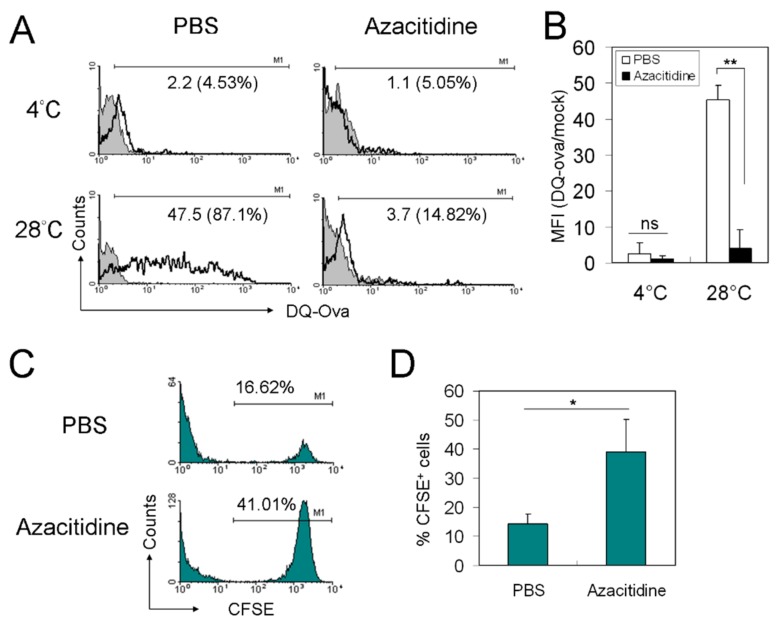
Impaired antigen processing and allogeneic rejection in azacitidine-treated zebrafish. (**A**) Plastic-adherent WKM cells from PBS- and azacitidine-treated zebrafish were incubated with DQ-Ova at 10 μg/mL for 2 h at 4 °C or 28 °C before being analyzed by flow cytometry. The average of three independent experiments are shown as mean ± standard deviation (SD) in **(B**). (**C**) CFSE-labeled ZLE cells (5 × 10^5^ cells per animal) were injected retro-orbitally into PBS- and azacitidine-treated zebrafish. After 24 h, the animals were sacrificed and their peripheral blood cells were analyzed by flow cytometry. The average of five independent experiments are shown as mean ± SD in (**D)**. * *p* < 0.05, ** *p* < 0.01, ns, not significant, by Student’s *t* test.

Next, we examined the functional significance of azacitidine-induced myelosuppression. Using a DQ-Ovalbumin (DQ-Ova)-based antigen processing assay, we found that adherent, kidney marrow myeloid cells from azacitidine-treated zebrafish have markedly impaired antigen processing *ex vivo*. At 2 h after adding the DQ-Ova dye (10 μg/mL) to the culture, the cells with released fluorescence as a result of endocytic protease cleavage comprised only 14.82% (mean fluorescence intensity (MFI) 3.7) of total cell population, compared with 87.1% (MFI 47.5) in the mock-treated control (Figure 3A). This difference remained statistically significant from the results of two additional independent experiments (Figure 3B). To test whether T cell function would also be affected, we injected carboxyfluorescein succinimidyl ester (CFSE)-labeled, allogeneic zebrafish liver epithelial (ZLE) cells via the retro-orbital route into the circulation of mock- and azacitidine-treated zebrafish (5 × 10^5^ cells per animal), and then measured the percentage of blood CFSE^+^ cells at 24 h post-injection to see if rejection occurred. Figure 3C,D show a ~2.5-fold higher percentage of CFSE^+^ cells in peripheral blood of azacitidine-treated zebrafish, compared with mock-treated control (14.27 ± 3.5% vs. 38.97 ± 11.3%, *p* < 0.05). Together, these findings indicate that T cells in azacitidine-treated zebrafish failed to effectively reject a *MHC*-mismatched allograft *in vivo*, which would otherwise take place in the context of functional antigen processing and presentation by myeloid antigen presenting cells (APCs).

### 2.3. Increased Susceptibility to Bacterial Infection in Azacitidine-Treated Zebrafish

Sepsis causes a high mortality rate in patients with high-risk MDS or AML receiving azacitidine treatment. Therefore, we tested whether azacitidine-treated zebrafish were more susceptible to bacterial infection than the mock-treated control. We chose three types of bacteria as our infectious agents. They are *E. coli*, a harmless normal flora, *Vibrio vulnificus*, a pathogen to both humans and sea creatures, and *Streptococcus iniae*, a pathogen highly virulent only to bony fish. 

**Figure 4:**
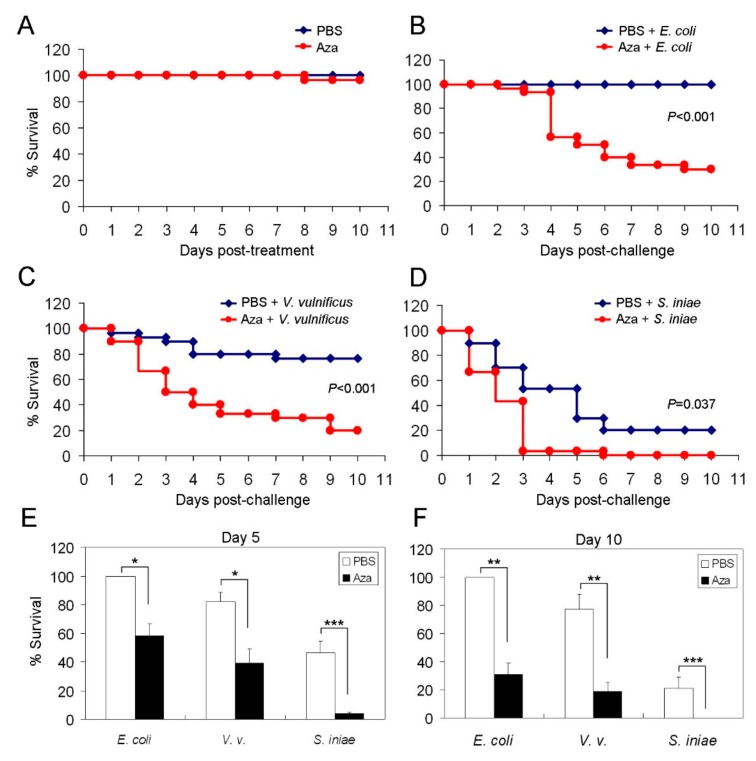
Increased susceptibility to bacterial infection in azacitidine-treated zebrafish. The Kaplan–Meier survival curve of zebrafish pretreated with PBS or azacitidine (Aza) (3 μg) for three days only (**A**), or followed by a challenge with (**B**) *E. coli* (10^7^ colony-forming units (CFU)), (**C**) *V. vulnificus* (10^5^ CFU) or (**D**) *S. iniae* (10^3^ CFU), *n* = 30 for each group, analyzed by the Kaplan-Meier log rank test. The Day-5 (**E**) and Day-10 (**F**) survival rates are shown. Results are mean ± SD of three independent experiments. * *p* < 0.05, ** *p* < 0.01, *** *p* < 0.001, by Student’s *t* test.

In our infection model, initially we demonstrated that three daily i.p. azacitidine (3 μg) administrations alone did not kill the animals, which remained alive for at least 10 additional days (Figure 4A). To test their susceptibility to bacterial infection, we injected the bacteria into mock- or azacitidine-pretreated zebrafish and followed up the survival of the tested animals for 10 days. The results of survival follow-up show a marked reduction in median overall survival (OS) in azacitidine-treated zebrafish vs. mock-treated control, when challenged with *E. coli* (5.5 d vs. >10 d, *p* < 0.001) (Figure 4B), *V. vulnificus* (3.5 d vs. >10 d, *p* < 0.001) (Figure 4C), or *S. iniae* (2.3 d vs. 4 d, *p* < 0.05) (Figure 4D). The 5- and 10-day survival rates of the azacitidine-treated group were also markedly lower than those of the mock-treated control (Figure 4E,F). Furthermore, we found that kidney marrow myeloid cells isolated from azacitidine-treated zebrafish had markedly impaired phagocytic activity *ex vivo* (Supplementary Materials Figure S1). Together, these results indicate that azacitidine treatment renders the zebrafish host highly susceptible to bacterial infection, even to the normal flora *E. coli*, likely because of impaired phagocytosis by the remaining macrophages in conjunction with impaired antigen processing. 

### 2.4. Increased Susceptibility to Bacterial Infection by Iron Overload in Azacitidine-Treated Zebrafish

**Figure 5:**
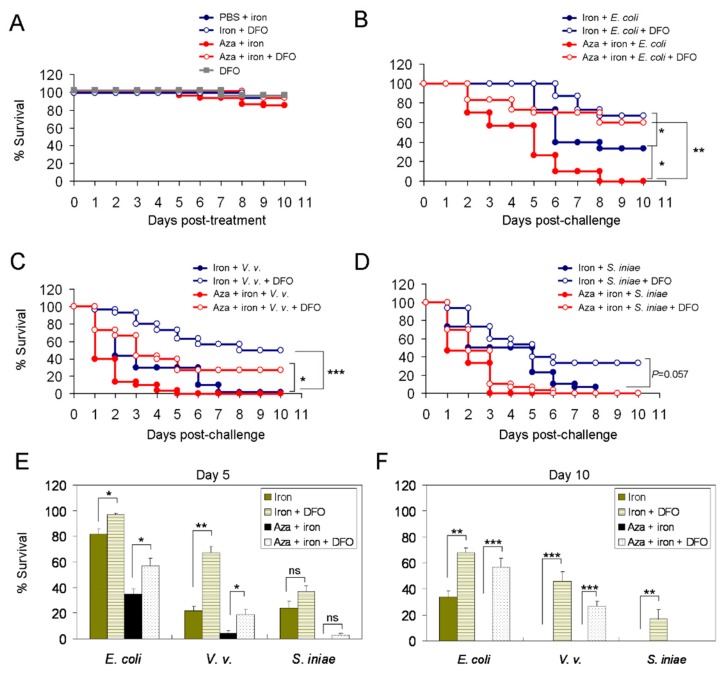
Increased susceptibility to bacterial infection in azacitidine-treated zebrafish in the presence of iron. The Kaplan–Meier survival curve of zebrafish pretreated with the indicated compounds, alone or in their combinations, for three days only (**A**), or followed by a challenge with (**B**) *E. coli* (10^7^ CFU), (**C**) *V. vulnificus* (10^5^ CFU) or (**D**) *S. iniae* (10^3^ CFU), *n* = 30 for each group, analyzed by the Kaplan–Meier log rank test. The Day-5 (**E**) and Day-10 (**F**) survival rates are shown. Results are mean ± SD of three independent experiments. * *p* < 0.05, ** *p* < 0.01, *** *p* < 0.001, ns, not significant, by Student’s *t* test.

Excess iron as measured by elevated serum ferritin levels is common in MDS patients because they need frequent blood transfusions. Since free iron is an essential component for bacterial growth, we tested whether iron overload could exacerbate bacterial infection in azacitidine-treated zebrafish. To this end, we included iron and its potent chelator deferoxamine (DFO) as two additional variables in our infection model. Figure 5A shows that either single treatment with iron (iron dextran, 50 μg), DFO (7 μg) or their combination with three prior daily azacitidine (3 μg) treatments could maintain a 92–97% survival rate of the tested animals on Day 10. Following a challenge with *E. coli* together with iron, the median OS dropped to 6 days (Figure 5B), compared to >10 days without iron in the mock-treated zebrafish (Figure 4B). In the azacitidine-treated animals, this *E. coli*-iron combination challenge further lowered the median OS to 5 days (hazard ratio (HR) = 2.41 [95% confidence interval (CI) 1.75–3.02], *p* < 0.05) (Figure 5B). Nevertheless, DFO could effectively reverse their vulnerability by extending their median OS to >10 days in both mock- and azacitidine-treated groups (HR = 0.475 [0.37–0.541], *p* < 0.05; HR = 0.311 [0.287–0.353], *p* < 0.01), indicating a pronounced iron-antagonizing effect (Figure 5B). When challenged with *V. vulnificus* together with iron, both mock- and azacitidine-treated zebrafish died quickly (median OS, 2 d vs. 1 d, *p* = 0.69) (Figure 5C). This suggests that acquisition of iron by *V. vulnificus* substantially augments its virulence [13], which outweighs azacitidine-induced myelosuppression. This dependence on iron was clearly indicated by the markedly prolonged survival in the mock-treated group (9 d vs. 2 d, HR = 0.251 [0.224–0.276], *p* < 0.001) and azacitidine-treated group (3 d vs. 1 d, HR = 0.413 [0.304–0.479], *p* < 0.05) in the presence of DFO (Figure 5C). At variance with these findings, iron did not significantly increase the virulence of *S. iniae*, and only a marginal DFO rescue effect was observed (Figure 5D). In the presence of iron, the 5-day survival rates of the azacitidine-treated group were also markedly lower than those of the mock-treated control following a challenge with *E. coli* and *V. vulnificus* (Figure 5E). The survival rates further dropped at Day 10, when the azacitidine-treated animals infected by either type of the bacteria all died (Figure 5F). Together, these results suggest that under azacitidine treatment, iron could exacerbate infection by *E. coli* and in particular *V. vulnificus*, but not that obvious to infection by *S. iniae* in zebrafish. 

### 2.5. Prolonged Survial to Bacterial Infection in Azacitidine-Treated Zebrafish Carrying the tp53^M214K^ Mutation 

**Figure 6:**
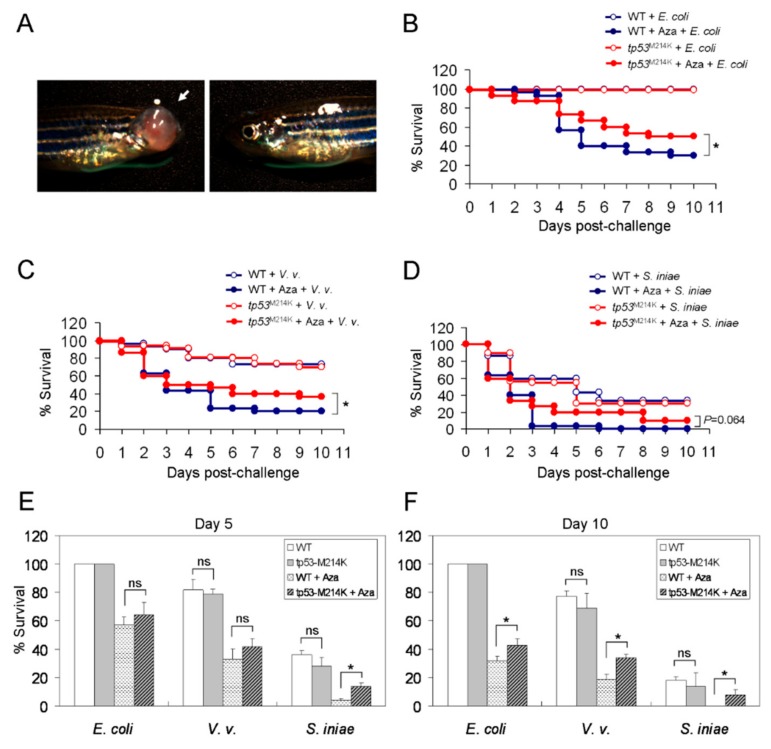
Prolonged survival to bacterial infection in azacitidine-treated zebrafish carrying the *tp53^M214K^* mutation. (**A**) Micrographs of an over 8-month-old *tp53^M214K^* mutant zebrafish showing zMPNST in its right eye. The Kaplan–Meier survival curve of mock- or azacitidine (Aza)-pretreated WT or *tp53^M214K^* mutant zebrafish challenged with (**B**) *E. coli* (10^7^ CFU), (**C**) *V. vulnificus* (10^5^ CFU) or (**D**) *S. iniae* (10^3^ CFU), *n* = 30 for each group, analyzed by the Kaplan–Meier log rank test. The Day-5 (**E**) and Day-10 (**F**) survival rates are shown. Results are mean ± SD of three independent experiments. * *p* < 0.05, ns, not significant, by Student’s *t* test.

The frequency of TP53 mutations is ~20% in MDS and ~8% in AML with a poor prognosis in spite of intensive chemotherapy. Taking advantage of an established tp53 mutant zebrafish line (*tp53^M214K^*) [14] (Figure 6A), we test the hypothesis that these animals may have defective hematopoiesis similar to MDS that results in impaired myeloid function. To this end, mock- and azacitidine (3 μg)-pretreated WT (*tp53^+/+^*) vs. tp53 mutant (*tp53^M214K/M214K^*) zebrafish were challenged with three types of bacteria and their survival was followed up for 10 days. Figure 6B shows that both mock-treated WT and *tp53^M214K^* mutant animals survived well following a challenge with *E. coli*. However, with azacitidine treatment, the WT animals died faster than the tp53 mutant counterparts (median OS, 5 d vs. 7.2 d, HR = 2.103 [1.685–2.462], *p* < 0.05). Similar results were obtained from the experiments challenged with *V. vulnificus* (median OS, 3 d vs. 4 d, HR = 1.927 [1.498–2.451], *p* < 0.05) (Figure 6C). At variance, following a challenge with *S. iniae*, both WT and tp53 mutant animals died quickly, although the latter survived somewhat longer on azacitidine with a difference not reaching statistical significance (*p* = 0.064) (Figure 6D). Furthermore, the analysis of 5-day and 10-day survival rates shows that the survival advantage emerged only after 5 days post-bacterial challenge in the azacitidine-treated group (Figure 6E,F). This suggests that *tp53^M214K^*-mediated resistance to lethal bacterial infection may be adaptive. The myeloid phagocytic function of the tp53 mutant kidney marrow cells and the subpopulations of the tp53 mutant kidney marrow and peripheral blood were not significantly suppressed compared to those of the WT following azacitidine treatment (Supplementary Materials Figures S1 and S2). Together, contrary to our hypothesis, *tp53^M214K^* did not impair myeloid function, but instead conferred some resistance to azacitidine-mediated myelosuppression.

**Figure 7:**
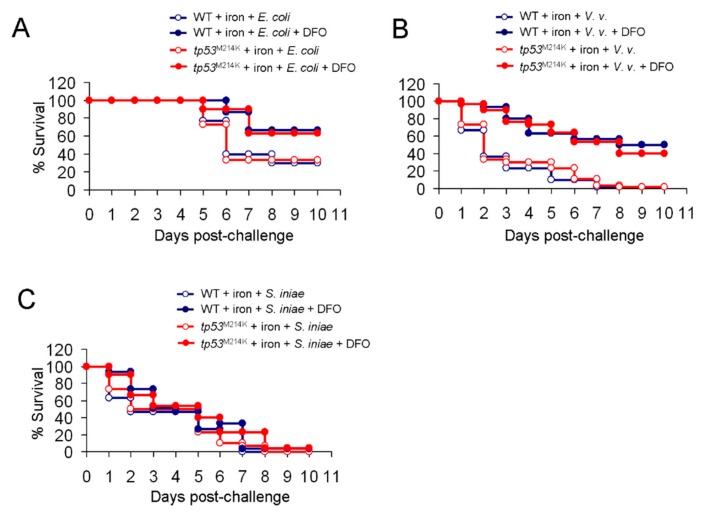
Survival to bacterial infection in iron-overloaded zebrafish carrying the *tp53^M214K^* mutation vs. the WT. The Kaplan–Meier survival curve of iron dextran (50 μg)-treated WT or *tp53^M214K^* mutant zebrafish challenged with (**A**) *E. coli* (10^7^ CFU), (**B**) *V. vulnificus* (10^5^ CFU) or (**C**) *S. iniae* (10^3^ CFU), with or without co-administration of deferoxamine (DFO, 7 μg), *n* = 30 for each group, analyzed by the Kaplan–Meier log rank test.

**Figure 8:**
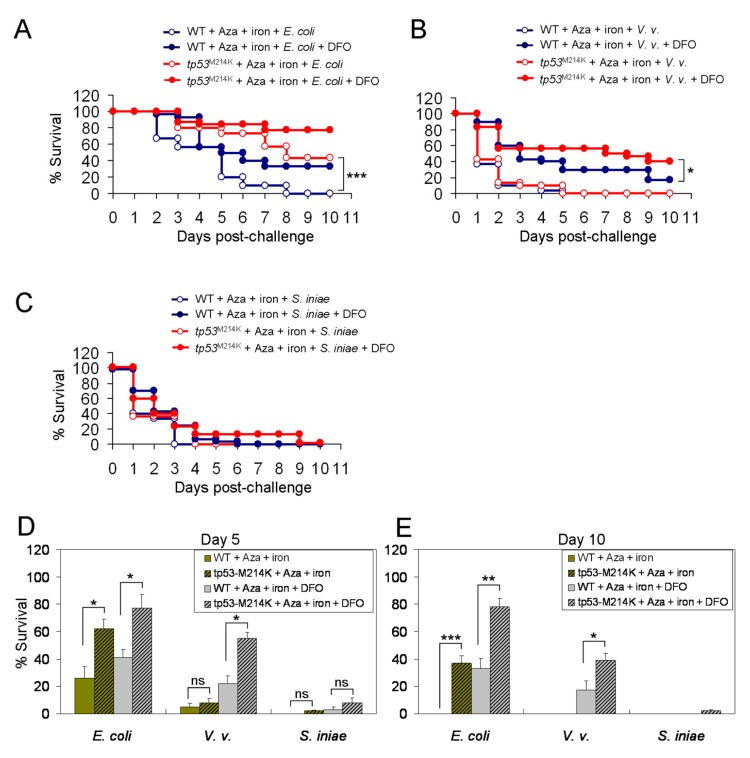
Prolonged survival to bacterial infection in azacitidine-treated zebrafish carrying the *tp53^M214K^* mutation irrespective of iron. The Kaplan–Meier survival curve of mock- or azacitidine-pretreated WT or *tp53^M214K^* mutant zebrafish challenged in the presence of indicated compounds together with (**A**) *E. coli* (10^7^ CFU), (**B**) *V. vulnificus* (10^5^ CFU) or (**C**) *S. iniae* (10^3^ CFU), *n* = 30 for each group, analyzed by the Kaplan-Meier log rank test. The Day-5 (**D**) and Day-10 (**E**) survival rates are shown. Results are mean ± SD of three independent experiments. * *p* < 0.05, ** *p* < 0.01, *** *p* < 0.001, ns, not significant, by Student’s *t* test.

We next examined whether *tp53^M214K^* could also confer protection from iron-mediated vulnerability to bacterial infection. We found that there was no reduced vulnerability of tp53 mutant zebrafish, vs. the WT, to infection by the three types of bacteria with iron overload (Figure 7A–C). However, when the animals were pretreated with azacitidine (3 μg) for three days, the median OS of tp53 mutant zebrafish was markedly longer than that of the WT (8 d vs. 5 d, HR = 0.293 [0.24–0.327], *p* < 0.001) following a challenge with *E. coli* (Figure 8A). DFO could effectively rescue only iron-mediated vulnerability (Figure 8A). At variance, when challenged with *V. vulnificus*, both WT and tp53 mutant zebrafish died very quickly with prior azacitidine treatment (Figure 8B). The protective effect of *tp53^M214K^* was seen only when iron was antagonized by DFO (Figure 8B) (median OS, 7 d vs. 3 d, HR = 0.583 [0.511–0.635], *p* < 0.05). On the other hand, no significant differences in survival were observed from the experiments challenged with *S. iniae*, no matter whether the animals were pretreated with azacitidine or not (Figure 8C). These findings were compatible with their 5- and 10-day survival rates (Figure 8D,E). Therefore, *tp53^M214K^* mutation again renders the zebrafish resistant to azacitidine-induced lethal bacterial infection. 

### 2.6. Prolonged Survival to Bacterial Infection in Azacitidine-Treated Wild-Type (WT) Zebrafish Reconstituted with the tp53^M214K^ Mutant Kidney Marrow

**Figure 9:**
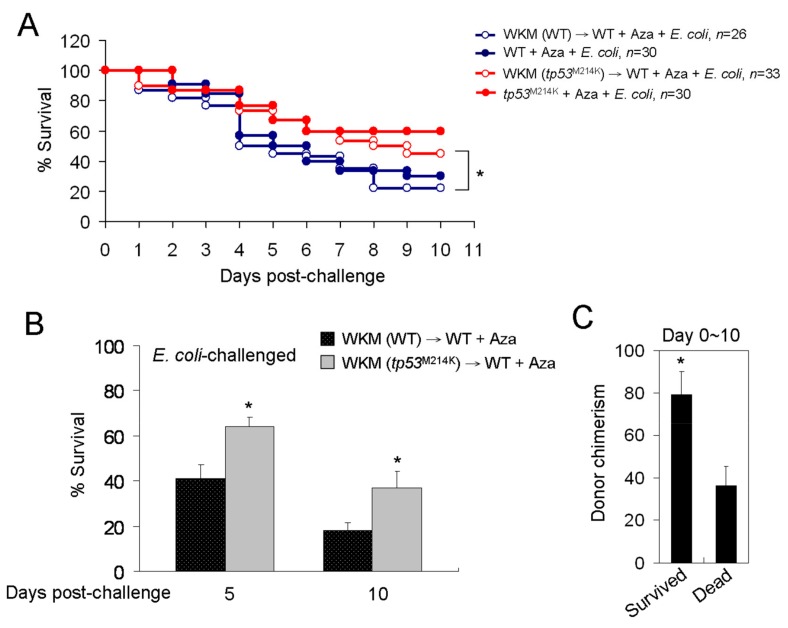
Prolonged survival to bacterial infection in azacitidine-treated WT zebrafish reconstituted with the *tp53^M214K^* mutant kidney marrow. The Kaplan–Meier survival curve of azacitidine-pretreated WT, *tp53^M214K^* mutant and their reconstituted recipients challenged with (**A**) *E. coli* (10^7^ CFU), analyzed by the Kaplan–Meier log rank test; * *p* < 0.05. The Day-5 and Day-10 survival rates are shown as mean ± SD of three independent experiments in (**B**); * *p* < 0.05, vs. WT WKM-reconstituted zebrafish. (**C**) The donor chimerism was determined by *tp53* genotyping of the *tp53^M214K^*-reconstituted WKM from survived and dead animals till Day 10. Results are mean ± SD of three independent experiments. * *p* < 0.05, vs. dead *tp53^M214K^*-reconstituted zebrafish.

To exclude the possibility that the observed *tp53^M214K^*-mediated effects were contributed by its own genetic background rather than the *tp53^M214K^* hematopoietic cells, we transplanted the *tp53^M214K^* mutant kidney marrow into the WT animals (*tp53^M214K^*→WT) to see if the WT recipient could survive longer when challenged with *E. coli*, following azacitidine treatment. Before transplantation, we treated the WT recipient with a single high dose of azacitidine (15 μg) instead of γ irradiation to achieve a myeloablation-like state based on our pilot study (data not shown). As a control, the WT kidney marrow cells were transplanted into the WT animals (WT→WT). The WT→*tp53^M214K^* transplantation was not performed because of the < 20% donor-recipient chimerism observed in our pilot study (data not shown). The animals that survived the transplantation for at least three months were used. Figure 9A shows that, when challenged with *E. coli* following azacitidine treatment, the WT zebrafish reconstituted with the *tp53^M214K^* kidney marrow survived longer than the WT kidney marrow-reconstituted counterparts (median OS, 8 d vs. 4.5 d, HR = 0.453 [0.412–0.507], *p* < 0.05). These results were compatible with their 5- and 10-day survival rates (Figure 9B). The donor chimerism, as determined by the presence of *tp53^M214K^* mutation in the recipient’s kidney marrow, was ~2-fold higher in the survived animals vs. the dead ones during the 10-day follow-up (Figure 9C). Therefore, the *tp53^M214K^*-mediated effects were not due to its general genetic background but was contributed by the *tp53^M214K^* hematopoietic cells.

### 2.7. Clinical Relevance of Azacitidine-Induced Myelosuppression and Iron Overload

**Figure 10:**
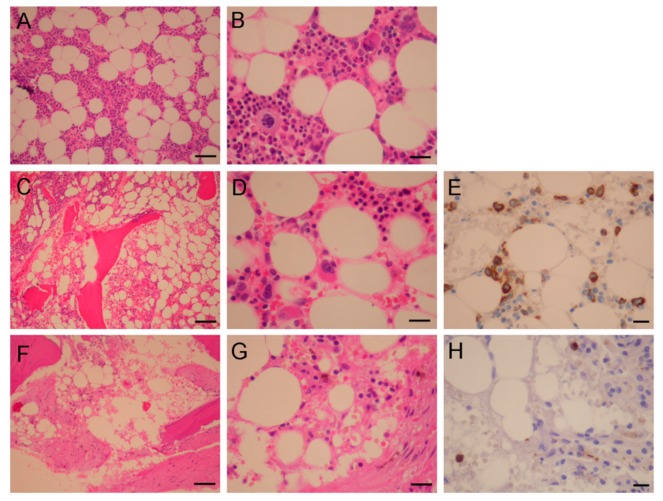
Severe bone marrow suppression in a myelodysplastic syndromes (MDS) case. Micrographs of bone marrow sections from normal (**A**,**B**) and a MDS case before (**C**–**E**) and after (**F**–**H**) azacitidine treatment, examined by Giemsa (**A**–**D**,**F**,**G**) and myeloperoxidase staining (**E**,**H**). Scale bars in (**A**,**C**,**F**), 100 μm; scale bar in (**B**,**D**,**G**,**E**,**H**), 10 μm.

For azacitidine-induced myelosuppression in the clinical practice, we present a case (75 years old) with high-risk MDS (RAEB-1) whose bone marrow had responded very well to azacitidine, but with pronounced cytotoxicity toward normal hematopoietic cells, in particular the myeloid series. Before treatment, as examined by histochemistry, the patient’s bone marrow showed mild hypocellularity with about 25%–40% of the marrow tissue (age-adjusted normal value: ~50% of the marrow tissue) [15] (Figure 10C). The myeloid/erythroid ratio was estimated to be 4–6:1 with markedly decreased erythroid series (Figure 10D). Myeloperoxidase staining revealed some early/atypical myeloid precursors with some dysplastic features (Figure 10E), while the lymphoid series appeared to be normal with mildly increased small lymphocytes and plasma cells (data not shown). After two cycles of azacitidine, the cellularity further dropped to < 20% with concomitant loss of many marrow units (Figure 10F). Small fragments of fibrotic tissue were noted focally in the marrow spaces with the presence of mildly increased reticulin in the marrow tissue (Figure 10F). The three major lineages of hematopoietic cells were further reduced in numbers with the erythroid series being < 10% of marrow cells (Figure 10G). Few myeloblasts were seen without a relative increase in the percentage of myeloid precursors (Figure 10H). The patient later progressed on subsequent azacitidine treatment and died of sepsis two months later. This case clearly shows a profound negative impact of azacitidine therapy early on hematopoiesis and host immunity.

**Figure 11:**
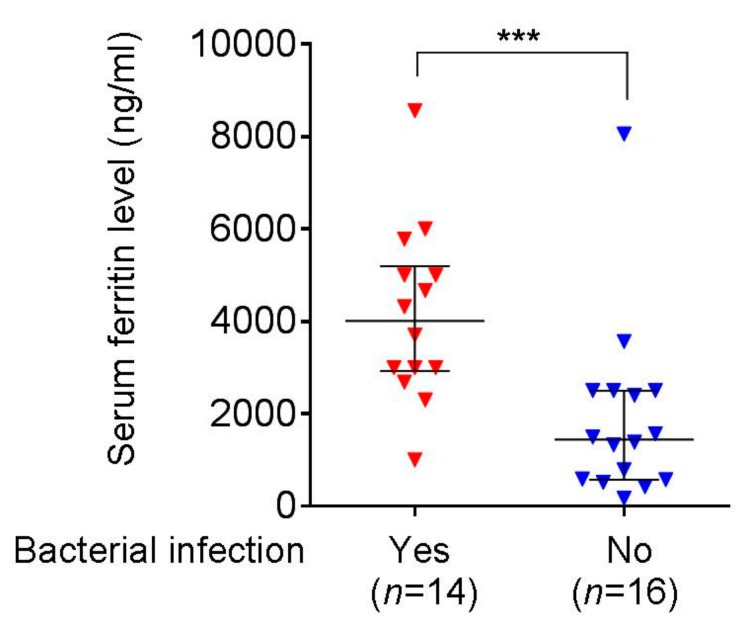
Serum ferritin levels in MDS patients with or without bacterial infection. Each symbol in the graph represents the data form each individual. The median and interquartile range are shown in lines. *** *p* < 0.001, by the Mann–Whitney *U* test.

As to the clinical relevance of iron overload, we analyzed the relationship between serum ferritin level and bacteremia in 30 patients with high-risk MDS receiving azacitidine treatment. Most of them required frequent blood transfusions to maintain the quality of life. Figure 11 shows that the median serum ferritin level was ~2.8-fold higher in patients with bacteria detected in their blood (or urine) compared to patients who had no detection of the microbes (4010 vs. 1441 ng/mL, *p* < 0.001; normal range, 12–300 ng/mL). Importantly, the vast majority of the bacteria identified were those frequently detected in opportunistic (normal flora) or nosocomial (*Pseudomonas* spp.) infection (Supplementary Materials Table S2). These findings are compatible with the results of our experiments which show that iron can exacerbate bacterial infection in azacitidine-treated zebrafish.

## 3. Discussion

In this study, we have for the first time explored whether the adult zebrafish can be utilized to investigate the hematological side effects of the hypomethylating drug azacitidine, which is currently used in the clinics for treating high-risk MDS and AML with lower-than-expected efficacy. In particular, we have investigated the azacitidine effect in the primary hematopoietic organ at the cellular and functional levels in healthy animals. This approach could reveal the drug effects alone on normal hematopoietic cells to avoid the interference of malignant hematopoietic cells, in particular the stem cells, with the interpretation of the results of analysis. 

In the AZA-001 trial, severe neutropenia (grade 3 or 4, i.e., > 50% or > 75% decrease from baseline) was observed in > 90% of patients receiving several cycles of azacitidine [7]. Compatible with this observation, we found an > 85% reduction in the myeloid series in the zebrafish kidney marrow (Figure 1B), following three-daily azacitidine doses, even at one fifth of the dose equivalent used in patients. This indicates that the normal zebrafish myeloid cell development was sensitive to this drug and was severely impaired by it. The less marked reduction (~13%) (Figure 1D) in the peripheral myeloid cell numbers may be due to their non-proliferating, terminally differentiated stage, which is still within their life span. On the other hand, the lymphoid cells were not significantly affected in numbers, only with a relative increase in the periphery observed, although their functional status was not clear. The red blood cells were also insignificantly influenced in numbers, but their size was significantly increased with a wide variation (Figure 1F,G and Figure 2A, right panel), a sign of compensatory erythropoiesis. The existence of the latter event was supported, but not proven, by an increase in early erythroid precursors, i.e., proerythroblasts and basophilic erythroblasts, but not in the late-stage precursors normoblasts (Figure 2D). The promyelocyte numbers were severely reduced by azacitidine (Figure 2D). These findings, together with an elevated extent of dysplasia and cell degeneration, suggest that this hypomethylating drug was exerting a cytotoxic rather than a cell- differentiating effect, similar to other DNA-damaging, chemotherapeutic drugs, e.g., cytarabine (ara-C).

As to the functional impact of azacitidine at the cellular level, first we showed that the zebrafish WKM-derived myeloid cells could be tested *ex vivo* in short-term using the techniques usually performed for mammalian cells to assess antigen processing. The ability of WKM-derived myeloid cells to process the model DQ-Ova antigen was remarkable at 28 °C in the control, but was almost abolished at 4 °C and in the azacitidine group at both temperatures (Figure 3A,B). Next, we showed that the short-term gentamicin protection assay could also be utilized to demonstrate impaired phagocytosis of *E. coli* by WKM-derived myeloid cells isolated from azacitidine-treated zebrafish, compared with the control (Supplementary Materials Figure S1). Furthermore, the retention of allogeneic ZLE cells in the zebrafish blood could be demonstrated using the CFSE-labeling technique, which showed that azacitidine-treated animals failed to reject this allograft effectively *in vivo* (Figure 3C,D). This allograft acceptance may be explained by azacitidine-induced impaired antigen processing, since allograft rejection often requires cross-presentation of allo-MHC antigen-derived peptides by myeloid dendritic cells (mDCs) to self-MHC-restricted CD8^+^ cytotoxic T cells (CTLs). However, in some cases, allograft rejection can occur through direct recognition of an intact allo-MHC by T cell receptor, when the former mimics the conformation of a self-MHC-peptide complex. In this scenario, impaired CTL function may also account for the allograft acceptance. Nevertheless, this possibility cannot be tested at the present time, because of the lack of appropriate tools and reagents to separate lymphocyte subsets in sufficient numbers for experiments. On the other hand, a recent study using an *ex vivo* human bone marrow cell co-culture system shows that azacitidne can affect hematopoiesis through influencing bone marrow stromal cells via essentially extracellular matrix molecules and interferon pathway components [16]. Whether this finding applies to zebrafish is not known at present and is worthy of further investigations. 

As to how azacitidine affects host immunity at the whole animal level, we established an infection model where the zebrafish were challenged with bacteria and their survival were followed up. The finding that azacitidine increased the susceptibility of zebrafish to infection by the normal flora *E. coli* was paralleled, but sadly, with the MDS case we presented here who had been hyper-responsive to azacitidine such that the bone marrow myeloid cells were almost wiped out following only several cycles of treatment, which resulted in fatal sepsis with opportunistic infection (Figure 10). In fact, this fatal side effect was clearly documented in the AZA-001 trial where the percent death was higher in the azacitidine group in the first three months than the conventional care group (11% vs. 9%) [7]. In addition, there were also more patients discontinuing azacitidine because of hematological toxicity than the control group within a year (5% vs. 2%) [7]. The reason why a fraction of patients had suffered from this fatal adverse event is not known. An urgent question to be addressed is to identify biomarkers that can predict the onset of fatal side effects for the best benefit of patients. This represents a crucial host variable that limits the azacitidine efficacy. With our zebrafish model, future work can be conducted to compare the genetic, epigenetic and transcriptomic profiles between the zebrafish that died early and those survived after azacitidine treatment.

When iron overload, a MDS-relevant condition, was included as a variable in our infection model, we found that the Gram-negative bacteria, *E. coli* and *V. vulnificus*, became more virulent (Figure 5), likely because of their iron dependence [17]. In particular, the latter bacterium has been shown by one of us to utilize iron as a growth booster *in vitro* and in mice [13], although the role of ferritin by itself as a host acute-phase protein in contributing to inflammatory processes cannot be excluded. Nevertheless, the role of bacterial iron-dependence was supported by the greatest extent of virulence attenuation by DFO in *V. vulnificus* in our infection model (Figure 5C). On the other hand, we did not observe marked iron dependence in the Gram-positive *S. iniae* (Figure 5D), unlike what was reported by Wang et al. [18]. Whether this was caused by strain differences is not known. In our pilot study, there was no death of iron-loaded zebrafish when challenged with as few as 500 colony-forming units (CFU) of *S. iniae* (data not shown), but the survival rate with or without iron was not significantly different when challenged with 1000 CFU (Figure 7C). Regardless, azacitidine plus iron still causes the worst survival in zebrafish challenged with the three types of bacteria. The clinical relevance was indicated in the markedly higher serum ferritin levels in azacitidine-treated MDS patients with bacterial infection compared with those without bacterial infection (Figure 11). Thus, uncontrolled iron overload may lead to higher chances of sepsis, representing another host variable that will compromise the azacitidine efficacy. 

The finding that *tp53^M214K^* could protect the zebrafish from azacitidine-mediated vulnerability to bacterial infection is intriguing. Our original hypothesis was to see otherwise, since *TP53* mutations occur frequently in MDS, and we reasoned that the tp53^M214K^ mutation may predispose the animals to MDS or AML with an impaired myeloid function that is sensitive to azacitidine. We performed this line of experiments using the tp53^M214K^ mutant zebrafish age 6~7 months before a fraction of them developed malignant peripheral neural sheath tumors (zMPNST) [14]. However, we did not observe cytopenia (Supplementary Materials Figure S2) or reduced myeloid phagocytic activity (Supplementary Materials Figure S1) in these *tp53^M214K^* mutant animals before and after azacitidine treatment. The preservation of myeloid function was further demonstrated by the improved survival of the *tp53^M214K^* mutant vs. the WT to bacterial infection following azacitidine treatment. These findings suggest that (i) the zebrafish *tp53^M214K^* mutation either causes tumorigenesis only in certain cell types or requires a long latency to promote hematologic malignancy, and (ii) the presence of this mutation protects hematopoietic cells from azacitidine-induced cytotoxicity without affecting their differentiation. This resistance to genotoxic stress was supported by the inability of zebrafish tp53^M214K^ protein to activate p21, resulting in the lack of G1/S arrest and subsequent apoptosis [14]. On the other hand, the site of *tp53* mutations may also determine its functional outcome. The tp53^M214K^ mutation in zebrafish was proposed to be orthologous to the *TP53^M246K^* mutation in humans. This mutation has been shown to destabilize TP53 by abolishing the hydrogen bonds between G245/M246 and R249 in the L2/L3 loops, resulting in loss-of-function of the protein [19]. However, the *TP53^M246K^* mutation was not reported in a clinical study enrolling 168 patients with MDS receiving treatment with azacitidine or decitabine [20]. This finding suggests that despite its loss-of-function at the molecular level, TP53^M246K^ does not play a major role in promoting the MDS phenotype in humans. Therefore, the interpretation of the results of our *tp53^M214K^* zebrafish experiments is limited to the resistance to azacitidine-induced genotoxic stress, as reflected by the unaffected hematopoiesis and myeloid function. The observation that the *tp53^M214K^* mutant myeloid cells have a survival advantage under azacitidine treatment can still explain the selective expansion of malignant MDS stem cells carrying *TP53* mutations in patients receiving this HMA [20]. Another interesting aspect of our study is the possible adaptive nature of tp53^M214K^-mediated resistance to lethal bacterial infection under azacitidine treatment. It is not known whether azacitidine could initially induce or upregulate some factors that are associated with tp53^M214K^ to preserve its WT function, but then loses this ability over time. It will also be worthwhile to test whether this putative adaptive nature also exists with other DNA-damaging drugs vs. drug without DNA-damaging activity.

From a clinical perspective, as implied by our *tp53^M214K^* zebrafish model, TP53 mutant MDS malignant stem cells may survive and expand at the expense of their TP53 WT normal counterparts under azacitidine treatment. This possibility is supported by the poor prognosis of MDS with TP53 mutations despite an initial response [20,21]. If *TP53* mutations represent a causal genetic variable affecting azacitidine efficacy, drugs capable of restoring TP53 function may improve the survival of MDS patients receiving this hypomethylating drug. In this regard, APR-246, a small molecule that promotes the refolding of mutant TP53 to its functional state, has been tested in combination with azacitidine in an ongoing phase II clinical trial, and the initial cohorts showed a high rate of response [22]. Lastly, since azacitidine and other HMAs, such as decitabine and guadecitabine, are also being tested in clinical trials for treating many solid tumor types (103 trials, according to https://clinicaltrials.gov/, using the drugs as search keywords), it would be pivotal to characterize the cellular and molecular basis of the response spectrum of these drugs using various experimental models combined with translational approaches for proper stratification of patients for their treatment in the real world in the near future.

## 4. Materials and Methods 

### 4.1. Zebrafish Maintenance, Bacterial Strains and Drugs

Adult zebrafish (*Danio rerio*) AB and embryos were maintained according to standard procedures at 28 °C [23]. A 10-h light/14-h dark day light cycle was used. The *tp53*^zdf1/+^ (AB) fish (Zebrafish International Resource Center, ZIRC) were in-crossed according to procedures reported by Berghmans et al. [14]. The genotype of the progeny was determined by restriction fragment length polymorphism (RFLP) (ZIRC) of the tail clip genomic DNA. The *zdf1* allele harbors a single T-to-A point mutation in exon 7 that changes Met to Lys at residue 214 which gives the genotype *tp53^M214K^*. The zebrafish *tp53^M214K^* mutation is an ortholog to the human *tp53^M246K^* (c.737T > A; p.M246K; exon 7). Only *tp53^M214K/M214K^* were used in this study. The study was approved by the Institutional Animal Care and Use Committee (no. 10476). *E. coli* DH5a (Yeastern Biotech, Taiwan) and *Vibrio vulnificus* YJ016 [13] were grown in shaking Luria-Bertani (LB) broth at 37 °C. The *S. iniae* wild-type strain 9117 [24] was cultured at 37 °C in Todd-Hewitt medium (Sigma-Aldrich, St. Louis, MO, USA) supplemented with 0.2% yeast extract (BD Biosciences, San Jose, CA, USA) and 2% proteose peptone (Sigma-Aldrich). Azacitidine (Vidaza^®^) was from Celgene (Summit, NJ, USA). Cytochalasin D, iron dextran and deferoxamine (DFO) were from Sigma-Aldrich. 

### 4.2. Flow Cytometric Analysis of Whole Kidney Marrow (WKM) and Peripheral Blood Cells 

The adult zebrafish kidney was surgically removed and made single cell suspensions by pipetting in 0.9× PBS with 5% fetal bovine serum (FBS) and 5 mM EDTA before being passing through a 40-μm mesh (BD Biosciences). Peripheral blood (1 μL) was obtained by cardiac puncture using a P10 pipet. WKM and peripheral blood cells were appropriately diluted with 0.9× PBS and subjected to analysis by the FACScalibur flow cytometer (BD Biosciences) with a linear forward scatter vs. log side scatter setting. Different subpopulations on the dot-plot were identified according to the work by de Jong et al. [25]. For determining hemoglobin (Hb) concentrations, mean corpuscular volume (MCV) and red cell distribution width-coefficient of variation (RDW-CV), peripheral blood was analyzed by the Sysmex XE-5000 automated hematology analyzer (Sysmex Europe GmbH, Germany). WKM and peripheral blood smears were stained with Liu’s stain solution (equivalent to Giemsa stain). 

### 4.3. DQ-Ovalbumin (DQ-Ova) Degradation Assay

DQ-Ova is a self-quenched conjugate of ovalbumin with BODIPY FL dye that exhibits bright green fluorescence upon proteolytic degradation which occurs in internalization and processing of exogenous antigens essentially in myeloid antigen presenting cells (Thermo Fisher). To perform this assay, plastic-adherent WKM cells (2 × 10^5^) were incubated with 10 μg/mL DQ-Ova (Thermo Fisher) in Dulbecco’s modified Eagle’s medium (DMEM) with 10% FBS for 2 h at 4 °C (inactivated) or 28 °C (activated) and then harvested for FACS analysis. It required 3–4 times more animals to obtain a sufficient number of cells from the azacitidine-treated group. 

### 4.4. Allogeneic Rejection Assay 

Zebrafish liver epithelial cell line ZLE [26] was labeled with CFSE using a CFSE-cell labeling kit (Abcam, Cambridge, MA) following the manufacturer’s instructions. CFSE-labeled ZLE cells (2 × 10^5^) were introduced into zebrafish circulation through retro-orbital injection with a 10-μL Hamilton syringe. The recipient animals were sacrificed 24 h later and their peripheral blood cells was analyzed by FACS. zMHC class I genotyping was performed by polymerase chain reaction (PCR) of genomic DNA with primers reported by de Jong et al. [25]. ZLE carries haplotype B (UBA^+^, UCA^+^), while zebrafish carry haplotype A/D (UFA^+^, UDA^+^, UXA2^+^). 

### 4.5. Gentamicin Protection Assay

Phagocytosis of live bacteria was performed as previously described [27]. Essentially, overnight cultures of bacteria were washed and resuspended in PBS to adjust concentrations. Plastic-adherent WKM cells (2 × 10^5^) were incubated with bacteria at a multiplicity-of-infection (MOI) of 10 for 45 min at 28 °C, followed by addition of gentamicin (100 µg/mL) and incubation for 20 min at 28 °C. Adherent WKM cells were harvested and spread on agar plates at serial dilutions to recover viable bacteria. CFUs were normalized to input bacteria and presented as a percent of the control group to compare the relative extent of phagocytosis. Cytochalasin D (10 μM) was used as a phagocytosis inhibitor. 

### 4.6. Bacterial Challenge and Survival Follow-Up

*E. coli*, *V. vulnificus* and *S. iniae* cultures were pelleted and resuspended with PBS to adjust concentrations for injection. *E. coli* (10^7^ CFU), *V. vulnificus* (10^5^ CFU) and *S. iniae* (10^3^ CFU) were injected i.p. into the zebrafish pretreated with three daily PBS or azacitidine (3 μg) doses. When needed, bacteria were mixed with iron dextran (50 μg), DFO (7 μg) or their combination for a single injection. The doses of bacteria and drugs were appropriately titrated in zebrafish as a pilot study before being used in the infection model. The azacitidine dose was calculated using the Mosteller’s body surface area estimation [28] with 75 mg/m^2^ daily dose per injection in patients as the reference. The standard treatment regimen for MDS is 75 mg/m^2^ s.c./day for 7 days (or 5-2-2) every 4 weeks [7]. In our study with zebrafish, the optimal regimen is one fifth (15 mg/m^2^) of the human equivalent daily dose for injecting zebrafish i.p. for three consecutive days. The concentration of each agent was appropriately adjusted so that the total injection volume would not exceed 10 μL. Thirty animals (age 6–8 months) per group were used. The deaths of the tested animals were counted every day up to 10 days. 

### 4.7. Whole Kidney Marrow (WKM) Reconstitution and Donor Chimerism

The recipient WT zebrafish were injected i.p. with high-dose azacitidine (15 μg) and then rested for three days. Subsequently, the donor WKM cells (5 × 10^4^) from *tp53*^m214k^ mutant or WT animals along with WT peripheral blood cells (2 × 10^6^) as carrier cells [29] were injected retro-orbitally into the tricane-anethesized recipients which were later kept for three months. Usually one third of the recipients survived this period. Next, the recipients were pretreated with azacitidne (3 μg) or PBS for three days before being challenged with bacteria, alone or in combination with other agents. Donor chimerism was determined by the detection of *tp53^M214K^* mutation, using the PCR-RFLP procedure (ZIRC), in the kidney marrow of *tp53^M214K^* WKM–reconstituted WT, survived and dead animals during the 10-day survival follow-up. 

### 4.8. Histology 

Bone marrow tissue specimens were decalcified and taken for section and then evaluated microscopically following Giemsa and myeloperoxidase staining. The use of patients’ medical records was approved by the institutional review board of Mackay Memorial Hospital (18MMHIS113).

### 4.9. Statistical Analysis

Statistical significance was assessed with Student’s *t*-test if the data of the two groups were normally distributed or the Mann*–*Whitney U test if they were not normally distributed; *p* < 0.05 was considered significant. Kaplan–Meier log-rank analysis was used to assess cumulative survival and was reported as *p* values and hazard ratios (HRs); *p* < 0.05 was considered significant. 

## 5. Conclusions

We have demonstrated that the zebrafish can be used as a tool to investigate hematological adverse events caused by a epigenetic cancer drug in detail. Using this animal model, we have revealed that azacitidine-induced myelosuppression and other host variables, iron overload and *tp53* mutations play an important role in determining the efficacy of azacitidine therapy, a standard of care regimen for the treatment of patients with MDS and AML.

## Supplementary Materials

The following are available online at https://www.mdpi.com/2072-6694/11/9/1294/s1: Figure S1: Impaired phagocytic activity by WKM-derived myeloid cells isolated from azacitidine (Aza)-treated wild-type (WT) zebrafish but not *tp53^M214K^* mutant zebrafish; Figure S2: Preservation of WKM myeloid cells from the azacitidine-treated *tp53**^M214K^* mutant zebrafish; Table S1: Marked azacitidine-induced myelosuppression in zebrafish whole kidney marrow (WKM) and peripheral blood as determined by flow cytometry; Table S2: Serum ferritin level, bacterial infection status and response to azacitidine treatment in patients with high-risk MDS.
