# Supplementary material for: The Impact of the Epigenetic Cancer Drug Azacitidine on Host Immunity: The Role of Myelosuppression, Iron Overload and tp53 Mutations in a Zebrafish Model
Source: Cancers (Basel). 2019 Sep 2;11(9):1294. doi: 10.3390/cancers11091294 (PMC6770285; doi:10.3390/cancers11091294)
Supplement: Supplementary file 1 [file cancers-11-01294-s001.pdf]

Supplementary Fig. S1

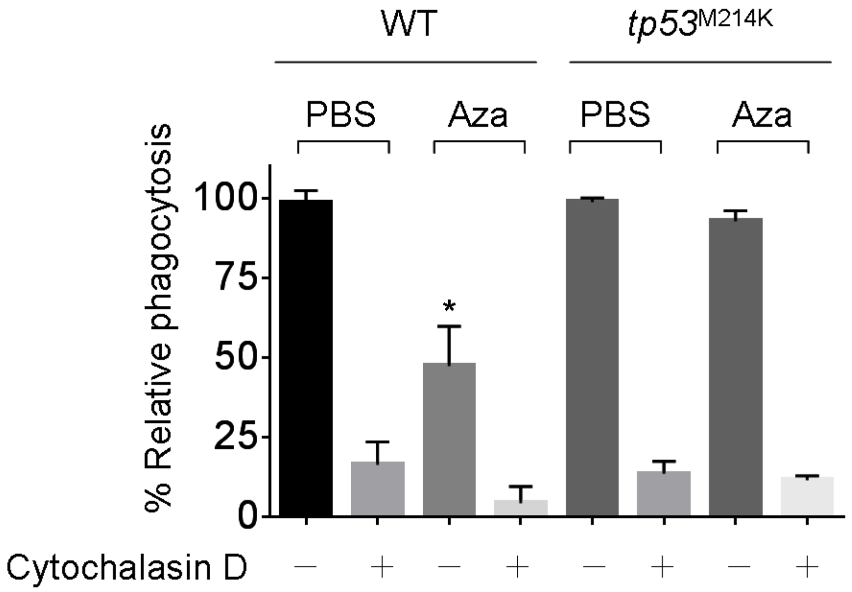

Figure S1. Impaired phagocytic activity by WKM-derived myeloid cells isolated from azacitidine (Aza)-treated wild-type (WT) zebrafish but not *tp53*^{M214K} mutant zebrafish. Plastic-adherent WKM cells (2×10^5) from PBS- or Aza-treated zebrafish were incubated with *E. coli* at an MOI of 10 for 45 minutes at 28°C with or without cytochalasin D (10 μM), followed by addition of gentamicin (100 μg/ml) and incubation for 20 min at 28°C. Adherent WKM cells were harvested and spread on agar plates at serial dilutions to recover viable bacteria. CFUs were normalized to input bacteria and presented as a percent of the PBS-treated group to compare the relative extent of phagocytosis. $n = 3$, * $p < 0.05$, *versus* PBS group without cytochalasin.

Supplementary Fig. S2

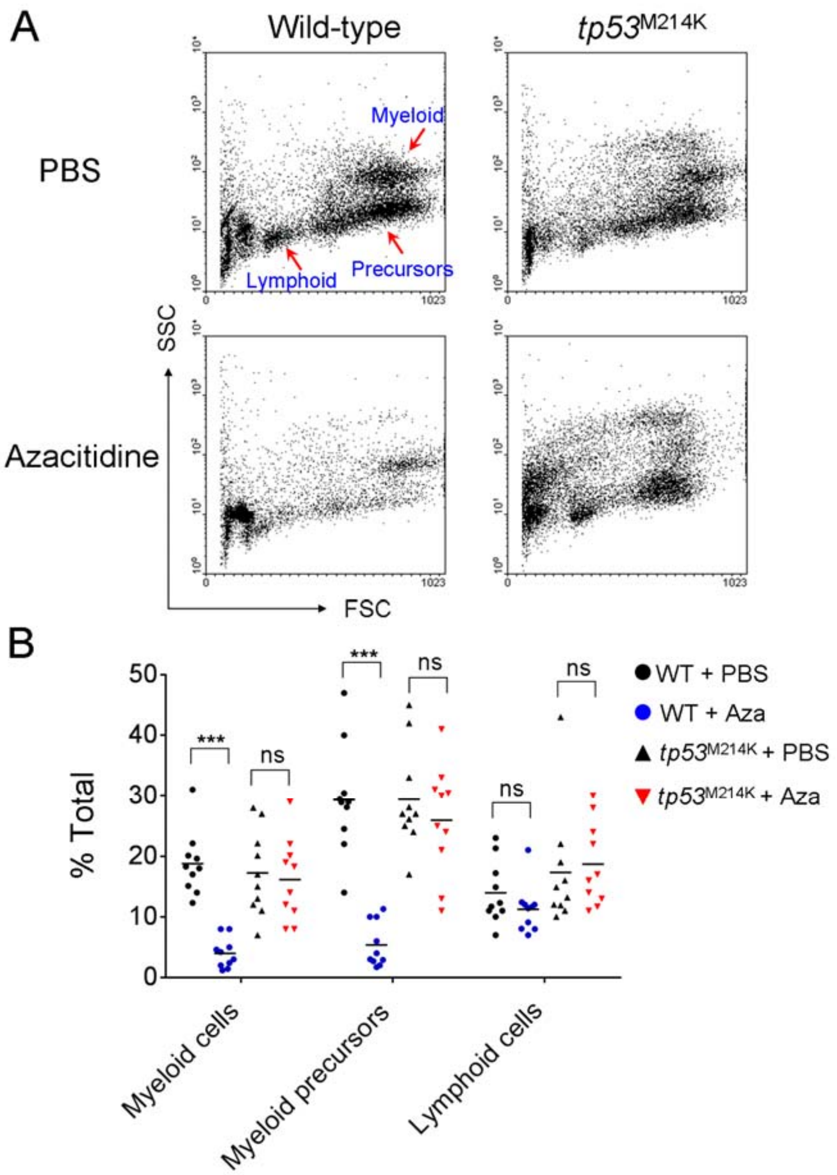

Fig. S2. Preservation of WKM myeloid cells from the azacitidine (Aza)-treated *tp53*^{M214K} mutant zebrafish. (A) Dot plots of WKM cells from wild-type and *tp53*^{M214K} mutant zebrafish treated with PBS or azacitidine (Aza) (3 μg) for three days. The WKM subsets are indicated each by an arrow with a label. (B) The relative percentage of WKM subpopulations as labeled were measured and presented as median in (B); each dot in the plot represents the percent gated area, n = 10, *** p < 0.001, ns, not significant.

Supplementary Table S1

Table S1. Marked azacitidine-induced myelosuppression in zebrafish whole kidney marrow (WKM) and peripheral blood as determined by flow cytometry

**Treatment**[a]	Mock		Azacitidine		
	Percentage	95% CI[b]	Percentage	95% CI	P value
**WKM**					
Myeloid cells	16.69	14.16-20.96	2.22	1.85-4.74	<0.001
Myeloid precursors	28.50	24.5-35.22	3.15	2.06-6.89	<0.001
Lymphoid cells	14.21	10.78-18.9	11.34	9.87-15.31	ns[c]
Erythrocytes	21.85	18.9-23.14	20.42	19.6-21.8	ns
**Peripheral blood**					
Myeloid cells	42.95	39.33-45.53	37.32	35.62-37.22	<0.001
Lymphoid cells	5.35	4.42-6.25	9.25	8.07-10.3	<0.05

[a]Wild-type zebrafish were mock-treated (1×PBS) or treated with azacitidine (3 μg/animal) in three consecutive days and their WKM or peripheral blood were collected on day 4 and analyzed, n = 10; [b] confidence interval; [c] not significant

Supplementary Table S2

Table S2. Serum ferritin level, bacterial infection status and response to azacitidine treatment in patients with high-risk MDS

Patient No.	Serum ferritin level (ng/ml)	Bacteria in blood	Response to azacitidine
1	8561	*Enterobact.*	Moderate
2	3000	*Enetrococcus*, *Acinetobact.*, *Pseudomonas* spp.	Poor
3	1382	No	Moderate
4	1000	[*Candida* spp. (urine)]	Good
5	8055	No	Good
6	4317	*Salmonella* spp.	Poor
7	5000	*Bacillus* spp.	Poor
8	4669	*E. coli*	Moderate
9	2500	No	Poor
10	1329	No	Poor
11	2300	*Acinetobact*. spp	Good
12	2500	No	Good
13	3000	*E. coli* (urine)	Good
14	3703	*M. tuberculosis*	Poor
15	1500	No	poor
16	2400	No	Moderate
17	2500	No	Moderate
18	587	No	Poor
19	3564	No	Poor
20	6000	*E. coli*	Good
21	5780	*Proteus* spp, *E. coli*	Poor
22	172	No	Moderate
23	789	No	Poor
24	3000	*Enterobacter*. spp	Poor
25	5000	*E. coli*; *Proteus* spp. (urine), *E. coli* (urine)	Poor
26	1570	No	Moderate
27	2688	*Enterococcus*, *Pseudomonas* spp.	Poor
28	423	No	Poor
29	519	No	Poor
30	572	No	Poor
